# Caught in the Whirl: A Case Report of Midgut Volvulus in an Elderly Patient

**DOI:** 10.5070/M5.52361

**Published:** 2026-07-31

**Authors:** Surina Khurana, Tien Lu, Carson Dangberg, Kristin Lewis

**Affiliations:** * University of California, Irvine, School of Medicine, Irvine, CA; ^ University of California, Irvine, Department of Emergency Medicine, Orange, CA

## Abstract

Midgut volvulus is a rare finding in adults, especially those without a known history of congenital gastrointestinal abnormalities. This is a case of a 78-year-old man who presented to the Emergency Department (ED) with two weeks of intermittent, postprandial abdominal bloating and pain. His examination was notable for tachycardia and mild abdominal distention. On computed tomography angiography (CTA), he was found to have midgut volvulus and underwent emergent exploratory laparotomy with Emergency General Surgery (EGS) where his bowel was found to be twisted clockwise around the mesentery. This case report highlights the diagnosis and treatment of a patient with the relatively uncommon diagnosis of midgut volvulus. In this report, we describe the patient’s presentation, important imaging findings, and key management principles for midgut volvulus.

**Topics:**

Volvulus, Midgut volvulus, abdominal pain.

**Figure:**
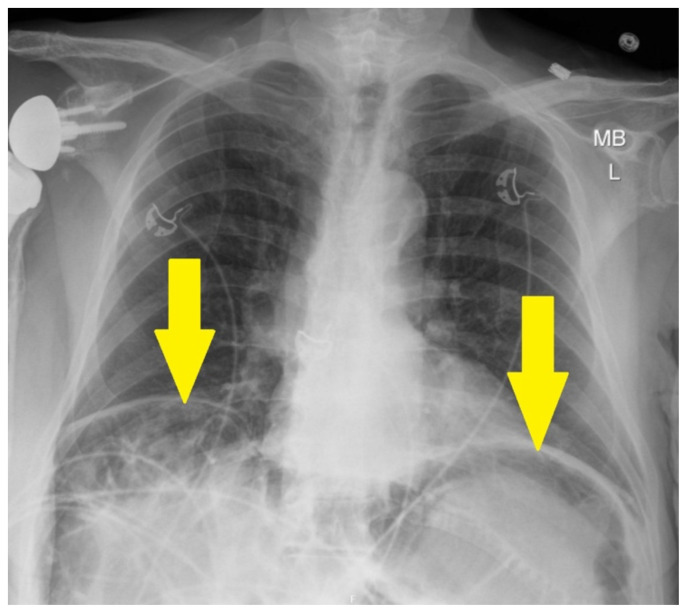


**Figure:**
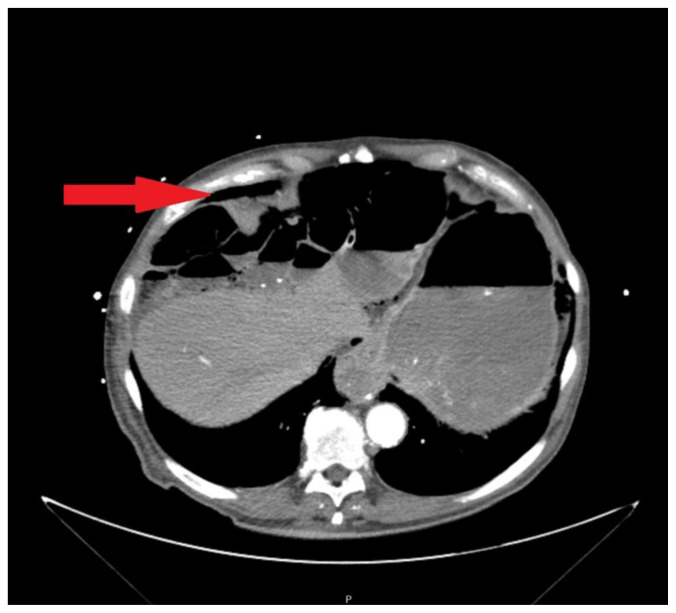


**Figure:**
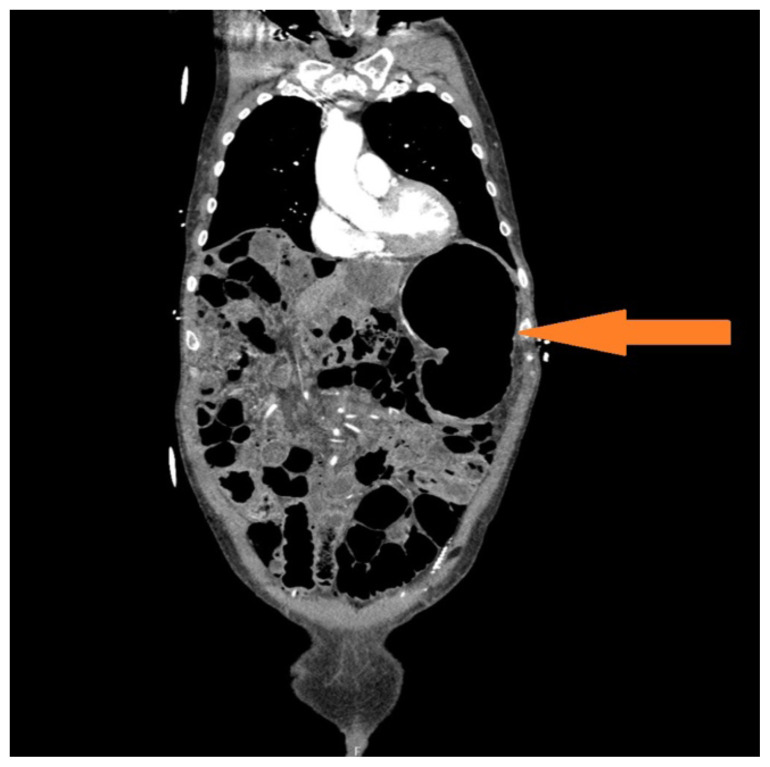


**Figure:**
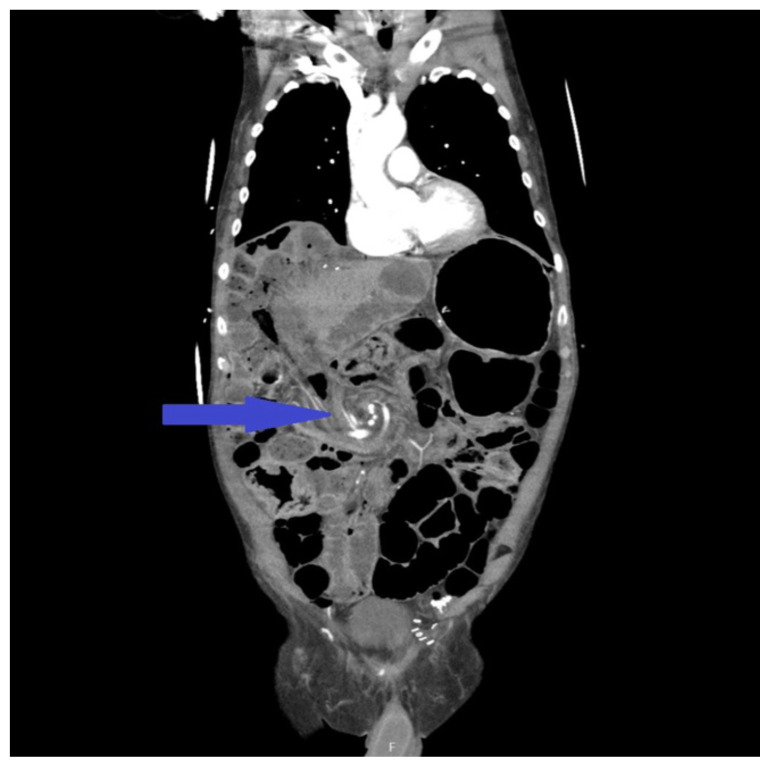


## Brief introduction

Midgut volvulus refers to a twisting of the small bowel that can lead to obstruction and compromised blood flow. It most commonly occurs in infants and young children, where it is frequently associated with underlying intestinal malrotation.^1^ It can also occur in adults, but this is extremely rare. Midgut volvulus is associated with several risk factors, including abdominal adhesions, pregnancy, endometriosis, and intestinal malrotation.^2^–^4^ Its prevalence varies geographically, with higher rates reported in the Middle East, Asia, and Africa, potentially related to dietary patterns, endemic infections, and socioeconomic factors.^2^,^5^,^6^ Adult midgut malrotation is rare, with an estimated incidence of 0.0001% – 0.2%, and approximately 15% of these cases present with midgut volvulus.^4^,^7^ The true incidence of midgut volvulus in adults is likely underestimated due to its intermittent and non-specific presentation, which may include abdominal pain, vomiting, and abdominal distension.^8^–^11^ Although reported mortality approaches 5%, this may be inflated by underdiagnosis; however, delayed recognition can lead to small bowel obstruction and ischemia.^1^,^10^ There is limited data on spontaneous resolution rates for midgut volvulus with intermittent symptoms. In a large U.S. based study, operative management was employed in 65.21% of cases versus nonoperative in 34.79%; non-operative management was a predictor of mortality.^12^ While definitive treatment is surgical, select patients with intermittent symptoms may be managed initially with close observation during further evaluation.

## Presenting concerns and clinical findings

A 78-year-old man with a past medical history of hypertension and gastroesophageal reflux disease presented to the emergency department (ED) with abdominal pain. He reported two weeks of intermittent postprandial abdominal bloating and pressure-like pain, along with several days of watery, non-bloody diarrhea. On the day of presentation, the pain acutely worsened after eating and was accompanied by nausea without vomiting. His daughter found his heart rate to be in the 130s–150s, prompting ED evaluation. He denied chest pain, palpitations, shortness of breath, fevers, chills, lower extremity swelling, history of blood clots, recent surgeries, or history of tobacco use. His surgical history was notable for cholecystectomy, and he had no known history of abdominal aortic aneurysm.

Initial vitals were remarkable for tachycardia to 150 beats per minute and elevated blood pressure at 170/114. He was afebrile with normal oxygen saturation on room air. On exam, he was alert and in no acute distress. Abdominal exam revealed a soft, mildly distended abdomen, without significant tenderness, rebound, or guarding. The remainder of the exam was normal.

## Significant findings

An upright chest x-ray demonstrated air underneath the diaphragm bilaterally (yellow arrows), enhancing the diaphragmatic contour. A computed tomography angiography (CTA) abdomen and pelvis showed scattered pneumoperitoneum (red arrow), dilated segments of small bowel (orange arrow), and clockwise swirling of the mesentery (blue arrow), concerning for mesenteric volvulus with possible bowel perforation. Labs showed a mildly low potassium, normal lactate, and mild leukocytosis.

## Patient course

Given the patient’s advanced age, significant tachycardia (150s), and history of intermittent postprandial abdominal pain with acute worsening, there was high concern for possible bowel ischemia, including pain out of proportion to examination, despite an initially reassuring abdominal exam. He was immediately placed in an ED bed for expedited evaluation. Point-of-care ultrasound was attempted to assess the abdominal aorta but was limited by overlying bowel gas. The patient received acetaminophen and hydromorphone for pain and a 1-liter intravenous (IV) Lactated Ringer’s fluid bolus. Given concern for mesenteric ischemia, a CTA of the abdomen and pelvis was prioritized along with laboratory studies and an upright chest x-ray, which revealed subdiaphragmatic free air concerning for bowel perforation. Emergency general surgery (EGS) was emergently consulted and evaluated the patient prior to transport to CT, while efforts were made to expedite imaging. On reassessment, the patient remained non-peritonitic, and his vital signs improved following fluid resuscitation. The decision to obtain expedited imaging was discussed with the surgical team. Because the patient’s tachycardia was improving with fluids and the patient was ready to be transported to CT at the time of evaluation, EGS requested the imaging be obtained prior to going to the operating room. Broad spectrum antibiotics were started for concern of bowel perforation.

CTA of the abdomen and pelvis showed pneumoperitoneum and swirling of the mesentery, concerning for mesenteric volvulus with bowel perforation. Laboratory studies showed a mild leukocytosis (11.6) and a normal lactate (1.4), which increased to 2.7 on repeat testing 90 minutes later despite receiving IV fluids. Acute coronary syndrome was ruled out. EGS recommended emergent operative intervention.

The patient underwent exploratory laparotomy with EGS, and his bowel was found to be twisted clockwise around the mesentery with some dilation and mild duskiness. Detorsion of the small bowel volvulus was performed with reassuring pink hue as a result. The bowel was run and did not show signs of perforation. An esophagogastroduodenoscopy and sigmoidoscopy were also performed immediately following detorsion without blood or perforation found. The EGS team hypothesized that that the free air seen on imaging may have been due to diverticula.

The patient was monitored for three days post-op without complication. He was discharged in stable condition. The patient has had three documented follow-up visits since discharge without any further complications.

## Discussion

Adult midgut volvulus is an uncommon but potentially life-threatening condition that often presents with nonspecific and intermittent symptoms, making diagnosis challenging. This case highlights the importance of maintaining a high index of suspicion in older patients with postprandial abdominal pain and physiologic abnormalities, even in the setting of a relatively benign abdominal exam.

Early diagnosis is paramount to prevent complications such as bowel necrosis and short intestinal syndrome, especially given that mortality rates approach 5%. Advanced imaging plays a critical role, particularly CT imaging. Characteristic findings include a “whirlpool” sign, or a swirled appearance of the mesentery and superior mesenteric vein (SMV) around the superior mesenteric artery (SMA), malrotated bowel configuration, transposition of the SMA/SMV, bowel obstruction, and pneumoperitoneum. The direction of the whirlpool swirl tends to be clockwise on ultrasound and counter-clockwise on CT.^3^,^13^ A systematic review of adult malrotation cases demonstrated that CT tends to be the most frequently used imaging modality in diagnosis, and the whirlpool sign was observed in roughly one third of cases. The most common imaging finding was abnormalities of the superior mesenteric axis.^14^

CTA of the abdomen and pelvis is the preferred imaging modality when bowel ischemia is suspected^12^ because it allows detailed evaluation of the mesenteric vasculature while still adequately assessing bowel pathology such as volvulus, which may present with overlapping clinical features. The sensitivity and specificity of the whirlpool sign on CTA in adults is 64–100% and 98–100%, respectively. When multiple CT predictors are present, such as multiple transition points, location, and whirlpool sign, specificity approaches 100%.^15^,^16^ CT is generally highly accurate for diagnosing small bowel obstruction and its causes, including volvulus.

This case highlights an uncommon presentation of midgut volvulus in an adult without typical predisposing risk factors such as extensive abdominal adhesions. The patient presented with non-specific symptoms, including postprandial abdominal pain, bloating, and nausea. Physical exam was notable for tachycardia, but was otherwise relatively benign, with minimal abdominal distension or tenderness. Thus, imaging was critical for timely diagnosis and guiding definitive management.

An additional complexity in this case was the presence of apparent pneumoperitoneum on imaging without an identifiable perforation at the time of surgery, despite running the bowel and performing an esophagogastroduodenoscopy and sigmoidoscopy. One possibility is the subdiaphragmatic lucency seen on the upright chest x-ray may have represented interposed bowel, as seen in Chilaiditi syndrome, rather than true free intraperitoneal air. Alternatively, transient or micro perforation, potentially related to diverticular disease, as suggested by the surgical team, may have sealed prior to operative exploration. These possibilities highlight the limitations of imaging in definitively distinguishing true pneumoperitoneum from mimics. The patient’s post-operative course was uncomplicated, and he was discharged home in stable condition. He has continued to do well with no complications noted at follow-up visits.

This patient’s case contributes to the literature on adult midgut volvulus due to malrotation, which is a rare but serious diagnosis. It also highlights the critical importance of imaging in diagnosis and management. Due to its high sensitivity for vascular compromise and characteristic signs, CTA is the preferred imaging modality for suspected volvulus in adults. However, adjunctive imaging such as upright abdominal or chest x-rays, which are useful for identifying free air, and abdominal ultrasound may be helpful in expediting the diagnosis and guiding early management, especially in unstable patients or resource-limited settings.

This case underscores several key principles: first, that normal laboratory values, including lactate, do not exclude early bowel ischemia; second, that a high index of suspicion should be maintained in elderly patients with postprandial abdominal pain and abnormal vital signs even when the abdominal exam appears reassuring, since the clinical history may suggest pain out of proportion to exam; and third, that timely CTA can facilitate rapid diagnosis and surgical management. Ultimately, early recognition and intervention are essential to prevent progression to bowel necrosis and other serious complications.

## Supplementary Information
















